# Patient Safety in Anticoagulation: Implementing Safety-II and FRAM in the Medical Curriculum

**DOI:** 10.5334/pme.2277

**Published:** 2026-04-09

**Authors:** Lauren S. Baidjoe, Liselotte M. van Dijk, Mirjam Simoons, An Tran, Marieke J. H. A. Kruip, Jorie Versmissen, Floor van Rosse

**Affiliations:** 1 Department of Hospital Pharmacy, Erasmus MC, University Medical Center Rotterdam, The Netherlands; 2 Program Healthcare Transformation, St. Antonius Hospital, Nieuwegein, The Netherlands; 3 Department of Pharmacy, Dijklander Hospital, Hoorn and Purmerend, The Netherlands; 4 Outpatient Pharmacy, Erasmus MC, University Medical Center Rotterdam, The Netherlands; 5 Department of Hematology and Department of Quality and Patient Care, Erasmus MC, University Medical Center Rotterdam, The Netherlands; 6 Department of Internal Medicine, Erasmus MC, University Medical Center Rotterdam, The Netherlands

## Abstract

Medication safety is a cornerstone of patient care, yet traditional error-focused approaches often limit learning opportunities in healthcare. While Safety-I focuses on identifying the root cause of the problem, Safety-II adds the dimension of adaptability and resilience of health care professionals in complex environments. This paper describes an innovative educational intervention aimed at integrating Safety-II, the Functional Resonance Analysis Method (FRAM) and anticoagulation management into an existing medication safety course within the Erasmus University medical curriculum. The course, designed for master’s-level medical students, uses Safety-II to analyze everyday clinical successes and to deepen understanding of anticoagulation processes in perioperative care. Students engage with clinical cases, create FRAM models, and reflect on their experiences during clerkships, which enhances their ability to recognize system strengths and adapt to complex situations. Teaching strategies such as ‘flipped classroom’ and ‘just-in-time teaching’ were incorporated, aligned with the medical curriculum, and enhanced active learning. Early implementation cycles led to iterative refinement of the course materials, informed by student and stakeholder feedback, as well as learning analytics. The course was successfully integrated into the medical curriculum, and our vision has since extended beyond the next generation of medical doctors. The Safety-II course has also been implemented into our cross-disciplinary education for healthcare professionals in specialist training. By integrating Safety-II thinking into various healthcare disciplines, we strive to contribute to a culture of safer, more resilient care, ensuring that adaptability and continuous learning are prioritized in all aspects of patient safety.

## Background & Need for Innovation

Medication safety remains a cornerstone of patient safety worldwide [1]. Historically, safety approaches have always focused on designing standardized protocols and systems to prevent failures. When an error does occur, we often find ourselves trying to identify a single root cause, asking questions such as ‘’Who caused this error?’’ or ‘’Which part of the protocol was insufficient?’’. This analysis typically results in the introduction of new rules, barriers, or protocols, aimed at preventing recurrence. This reflects the Safety-I perspective [2, 3].

Safety-I assesses safety in healthcare through the absence of harm, using metrics like incident rates and adverse event rates. Interventions that follow, can often oversimplify the complexities of clinical care and limit opportunities for learning from daily practice [2, 3].

The Safety-II approach recognizes that safety emerges not from rigid adherence to protocol but from the human capacity to adapt, apply creativity and make flexible and goal-oriented decisions in complex environments, thereby fostering resilience in everyday practice [3, 4]. By studying everyday performance, reflecting on all processes, and not just the ones that fail, Safety-II highlights hidden strengths, recognizes daily successes and creates opportunities to learn from daily practice [5]. The Functional Resonance Analysis Method (FRAM) supports Safety-II thinking by comparing the Work as Imagined (WAI, the process according to protocols or instructions) with the Work as Done (WAD, the process as it occurs in daily practice) [6].

While Safety-II thinking and FRAM have been successfully applied in high-risk, complex industries like aviation, healthcare practice still predominantly relies on traditional Safety-I approaches, with Safety-II principles being integrated only gradually. Yet, the complex nature of healthcare practice requires continuous adaptation and resilience, making FRAM a valuable tool to support reflection on daily processes [7]. Anticoagulation management within perioperative care processes exemplifies this complexity and the high risks involved: it requires multidisciplinary collaboration between specialists, nurses, pharmacists, and general practitioners, demands decisions on treatment for the individual patient, and involves navigating varying guidelines and protocols, all ultimately aimed at balancing thromboembolic and bleeding risks [7, 8]. Despite protocols and safety measures, numerous Safety-I-focused studies show perceived difficulties with anticoagulant and antiplatelet therapy. For example, the HARM-study found these therapies to be among the top contributors to medication-related hospital admissions, accounting for 15% of potentially preventable cases [9, 10]. Additionally, the World Health Organization’s Global Patient Safety Action Plan 2021–2030 states that medication errors remain a leading cause of preventable patient harm, resulting in a global patient safety initiative ‘’Medication Without Harm’’ [1]. Moreover, higher error rates on exam questions among medical students, combined with existing Safety-I-focused medication safety classes in the medical curriculum, led us to innovate medication safety education in the master’s program by integrating Safety-II thinking [11].

In medical education in the Erasmus Medical Center, medication safety courses primarily focus on error prevention and root cause analyses, reflecting Safety-I. Given the existing challenges in anticoagulation management and the limitations of traditional error-focused safety education, we saw an opportunity to transform a medication safety class in the master’s medicine curriculum into an innovative Safety-II-course. By integrating Safety-II, the FRAM method and clinical anticoagulation management, the course emphasizes not only reflecting on the occurrence and causes of errors (Safety-I), but also analyzing everyday clinical successes and adaptations, while simultaneously deepening students’ knowledge of anticoagulation management.

## Goal of Innovation

The goal of innovating this course is to achieve a shift in mindset amongst medical students, studying to become medical doctors, to view safety as a variable, resilient and integral part of healthcare. Through the introduction of Safety-II and FRAM, we encourage students to develop a ‘helicopter view’ of clinical workflows, while also deepening their understanding of the high-risk process of anticoagulation management. By focusing on adaptability to variability, we hope to cultivate critical thinking, recognition of system and human strengths, and a broader perspective on patient safety. Aligned with the medical curriculum at Erasmus University, the course incorporates innovative teaching strategies, such as the flipped classroom and just-in-time teaching, to foster active learning, engagement, and reflection.

## Steps taken for Development & Implementation of Innovation

### Curriculum description

The medical curriculum at Erasmus University in Rotterdam, The Netherlands, integrates medication safety education into both the bachelor’s (undergraduate) and master’s (graduate) curriculum, as part of clinical pharmacotherapy (CPT) education. During the 3-year bachelor’s program, students primarily focus on pharmacology and pharmacotherapy, with an introduction to the Safety-I principle. In their 3-year master’s program, they are trained to apply this knowledge during several clerkships, each preceded by a theoretical instruction block. The Safety-II principle is introduced in their fourth year of medical school, prior to their surgical clerkship, as this is when students are most likely to encounter patients using anticoagulants, making it an ideal moment to integrate Safety-II and anticoagulation management. The surgical clerkship is only their second clerkship, meaning students have limited practical experience, although they might have some from student jobs. Every ten weeks, a new group of approximately 72 master’s students participates in this Safety-II course.

### Course development

We assembled a multidisciplinary team consisting of medical specialists, clinical pharmacology teachers, (hospital) pharmacists, quality and educational advisors, and an external party that developed the e-learning module and provided learning analytics.

First, we determined the learning objectives for this course. By the end of the course, students are expected to understand and begin practicing Safety-II thinking, gaining insights into what a FRAM model portrays and how they can use it to analyze work processes. Concurrently, we developed three representative clinical cases focusing on perioperative anticoagulation management:

- A patient on anticoagulants is electively admitted.
- A patient on anticoagulants is admitted urgently.
- A patient on anticoagulants is discharged.

The course design followed the Learn–Practice–Apply–Review (LPAR) method, which guided the design, development, and evaluation of our innovation [12].

We introduce students to Safety-II and FRAM through an e-learning module, following the principles of a flipped classroom. In this preparatory module, students **learn** about Safety-II and the FRAM research method, supported by formative quizzes. Our learning objectives and exemplary questions can be found in Table 1. During the subsequent in-class session, just-in-time teaching is applied: students recap and reflect back on the materials, allowing the teacher to address gaps in their understanding.

**Table 1:** Learning objectives (LO) and exemplary questions in e-learning module.


LO1: THE STUDENT KNOWS THE DIFFERENCE BETWEEN SAFETY-I AND SAFETY-II	LO2: THE STUDENT KNOWS WHAT THE FRAM METHOD IS AND CAN APPLY THIS TO A CLINICAL CASE

**1. The Safety-II approach focusses on…** a*. Opportunities and possibilities* b. Personnel and protocols c. National procedures and incidents d. The Hospital Admissions Related to Medication (HARM) study and re-hospitalizations	**1. In the context of medication safety, what does the term “Work-as-imagined” mean in comparison to “Work-as-done”?** a. WAI refers to choosing treatment based on guidelines and protocols, whereas WAD refers to situations where medications work effectively and without complications. b*. WAI represents the prescribed protocols and procedures that should be followed, while WAD represents the way healthcare providers actually work in practice, taking into account the complexity and variability of situations*. c. WAI is the way pharmacists prepare medications, while WAD is the way patients actually take the medications.

**2. Which of the following research methods fits a Safety-I approach?** a*. Prevention and Recovery Information System for Monitoring and Analysis (PRISMA)* b. Functional Resonance Analysis Method (FRAM)	**2. Matching question** Students match aspects to a function in a clinical case. For example: In case of ‘’A fatigued patient’’, students determine whether the aspect ‘’No fear of injections’’ should be classified as an Input, Output, Precondition, Resource, Control or Time requirement for the function ‘’Referring a patient for blood tests”.


**Figure 1:**
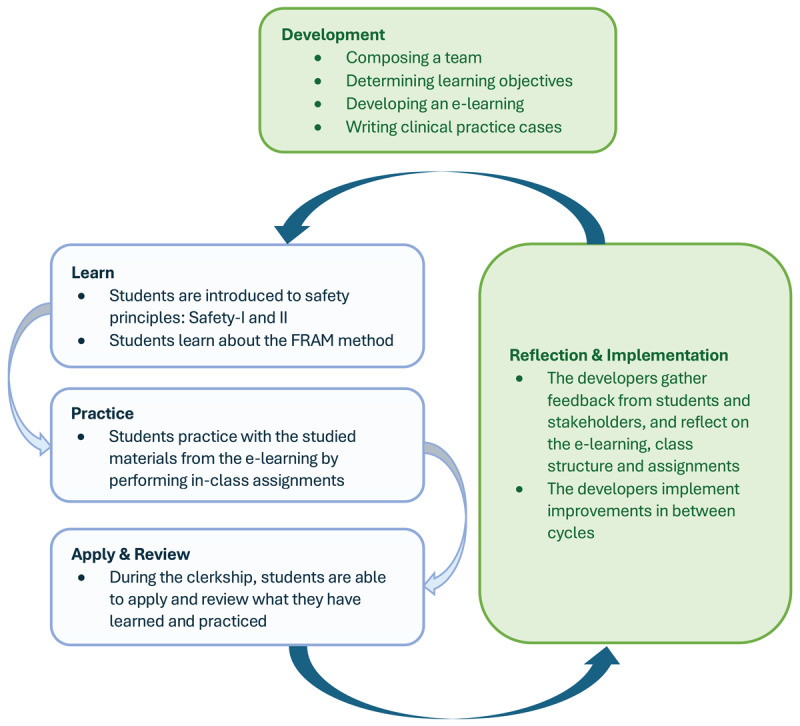
**Design cyclical action research**. A visual representation of the continuous learning cycle that was applied during the development of the innovative medication safety course in the master’s medicine curriculum. *Green: actions by the developers; Blue: actions by the students*.

Students then **practice** with clinical cases in small groups, constructing a Work as Imagined FRAM based on guidelines for a patient on anticoagulants who is electively/urgently admitted or discharged. This exercise involves creating a process sketch, gaining insights into who is involved at each stage, and how the individual roles are interdependent. Afterwards, the students present their analyses in class and receive feedforward to apply during their upcoming clerkship. During their clerkship, students are encouraged to **apply** gathered knowledge and list observations regarding patient and medication safety. Post-clerkship students **review** their experiences in light of Safety-II and FRAM. The course was iteratively refined based on learning analytics and feedback from both students and stakeholders, as visualized in Figure 1.

## Evaluation of Innovation

Through Safety-II education we aim to show students that deviation from a protocol or original plan may reflect resilience rather than failure. During the development of this course, we, as developers, experienced a similar reality: the course we developed, our Work as Done, differed from the initial course design, our Work as Imagined.

For example, we introduced the FRAM method in the e-learning through a simplified example: ‘’How to plan a FRAM-ily dinner’’. However, students indicated that this example was too abstract for an already complex research method. This was reflected in the learning analytics: 0% of the students completed the FRAM-ily dinner exercise successfully on their first attempt. In response, we developed two clinical e-learning cases, focusing on topics more relevant to their field, such as postoperative wounds and orthopedic procedures. This approach helped students engage more effectively with the FRAM method. Learning analytics showed improved first-attempt success rates of 45% and 34% for the clinical exercises, respectively.

Learning analytics also revealed that while the clinical cases posed some challenges, students generally performed well. For example, in the matching task described earlier, students (N = 127) matched a case description (e.g., next injection in 4 hours) to the correct FRAM-aspect, most students answered correctly (N = 102), although some struggled with the question and selected the wrong answer (N = 20).

Moreover, after the first teaching cycle, stakeholder feedback indicated that the in-class assignment, Clinical Case 3: “A patient on anticoagulants is discharged,” lacked specificity due to variations in actual discharge processes. Consequently, we split it into two separate cases:

- A patient on anticoagulants is discharged home.
- A patient on anticoagulants is discharged to a nursing home.

As a final assignment, we asked students to create a “Work as Done” FRAM for an observed process during their clerkship and discuss it with their supervisor. However, students experienced this assignment as uncomfortable, as it required them to potentially critique processes at a clerkship site as guests. Therefore, we redesigned the assignment into a reflection class. This change provided a safe space for students to share their clerkship experiences and observations, while still focusing on safety management. With this adjustment, we aimed to create a safe, less confrontational learning environment.

After completing the educational week and subsequent clerkship, we conducted interviews with several students to assess whether they had adopted the Safety-II perspective. They noted becoming more attentive to care processes and creative solutions that enhanced clinical care, instead of just focusing on mistakes or inconsistencies. Additionally, students mentioned they started to develop an overview, which provided insights in the way various professionals interact within a complex system.

One year after full implementation, we piloted an innovative addition: an electronic prescribing exercise using the EDUPS platform, a platform which mimics a real prescribing system. This addition aligned with students’ clinical interest and their future role as prescribers, while also broadening the focus beyond procedural analysis. It allowed them to apply pharmacotherapeutic reasoning in a systems context, reinforcing both medication safety principles and the practicalities of prescribing. Qualitative student feedback indicated high engagement and clear added learning value, which supported continued integration of the EDUPS platform in future iterations of the course.

## Critical Reflection

### Development

Using the backwards developing strategy, we constructed learning objectives prior to study materials and assignments, which contributed to the alignment of these components. Through application of the LPAR approach, we deepened student engagement by guiding students step-by-step from knowledge acquisition to application and reflection. We also consider the flipped classroom model valuable as it enabled students to enter class with prior familiarity with the subject, allowing for more in-depth and active learning in class.

Aligned with findings by Birkeli et al. (2025), we recognized that working within a multidisciplinary team contributed to the course quality [13]. Close collaboration with stakeholders through frequent meetings enabled the development of engaging and realistic clinical cases for students to explore.

We also learned that it is crucial to develop educational materials with an appropriate level of difficulty. Relying on first instincts and creating simplified assignments proved insufficient to achieve the desired learning outcomes. As suggested by Kelekar et al. (2020), the use of clinical cases prior to clerkships enhances opportunities to emphasize key concepts in clinical reasoning [14].

### Implementation

Implementation showed that our educational materials required adaptation once put into practice. Feedback from students and stakeholders led us to iteratively refine the e-learning, class structure, and assignments. This cycle of implementation, evaluation, and rapid adjustment strengthened both the relevance of the course materials and student engagement.

During implementation, we learned about the importance of engaging with the target audience. By tailoring our delivery and providing clear guidance, students’ enthusiasm for exploring new theories and analytical methods increased, as their initial resistance diminished. However, we approached student feedback with caution, acknowledging that individual interpretations of course elements may differ. As course developers, we recognized that it is impossible to accommodate every student’s preference, which emphasizes the need to focus on core learning objectives.

Verhagen et al. describe several challenges in implementing Safety-II principles in healthcare, such as the difficulty of “moving beyond system descriptions towards actionable interventions’’ [3]. This was also apparent in our teaching practice. While students were able to construct a Work as Imagined FRAM, interpreting these visualizations and translating them into improvements proved more challenging. This finding highlights a broader limitation of FRAM itself: despite its descriptive power, it provides limited guidance on translating insights into concrete interventions [3]. Bridging the gap between system understanding and practical application remains a key challenge in operationalizing Safety-II, a challenge we aim to address through the implementation of the EDUPS prescribing exercise.

Nonetheless, the overall implementation proceeded smoothly, likely because the course already had a predefined place in the curriculum. By innovating an existing class rather than introducing a new element, we avoided the typical resistance and logistical challenges often encountered in curriculum innovations.

### Evaluation

Our reach has been extensive. Across six course cycles, we taught 21 student groups, each consisting of approximately 25 students. Learning analytics were collected from our custom e-learning module and the in-class assignments yielded a total of 64 student-created FRAM models which were used to assess students’ understanding, the effectiveness of our teaching methods and opportunities to refine the course.

Through continuous evaluation and course refinement, teachers reflected on their teaching style and consequently improved their delivery. The impact of these improvements was evident in the FRAM models produced by students. **Appendix 1** shows three FRAM models, representing student analysis: a basic model from cycle 1 and two very extensively thought-out models from cycle 4 and cycle 6. The models shown are recreated in Microsoft Visio, based on the original FRAM models created by students.

Based on our in-class observation that students struggled to connect course content to clinical practice, a challenge also noted by Birkeli et al. (2025), we piloted the addition of a prescribing assignment in EDUPS [13]. Although not explicitly designed for the Safety-II and FRAM class, this task encouraged students to adopt a broader perspective on medication safety. While prescribing, students considered what information the next healthcare professional would need, fostering anticipatory thinking and continuity of care.

Notably, having an expert teach the class and lead the in-class assignment also contributes to the learning effect, allowing students to understand anticoagulation management with the broader workflow perspective gained from constructing a FRAM.

Finally, the evaluation highlighted both the course’s strengths and its broader potential. While constructing a FRAM, students were also encouraged to reflect on the roles and responsibilities of healthcare professionals they might not usually consider. Furthermore, beyond its successful integration into the medical curriculum, the course has now also been embedded in the cross-disciplinary education program for healthcare professionals in specialist training.

**What we’ve learned from our Work as Done**

**Table:** 

*Development*	1. Involve stakeholders with diverse backgrounds
	2. Compose a list of learning objectives prior to study material or assignments
*Implementation*	3. Listen to wishes of the target group
	4. Create a safe environment for students to reflect on their clerkship
	5. Be prepared and willing to adapt the course in real time based on student and stakeholder feedback
*Evaluation*	6. Create a continuous reflection – improvement loop to improve the course
	7. Try out Safety-II goggles yourself and explore options for wider implementation!

## Appendix 1. FRAM models developed by students

*a. FRAM model from cycle 1 (May 2023); b. FRAM model from cycle 4 (December 2023); c. FRAM model from cycle 6 (May 2024). The visualizations shown are recreated in Microsoft Visio based on the original student models*.

a. The figure above shows a basic FRAM model developed during cycle 1, illustrating perioperative anticoagulation management process prior to a scheduled admission. Notably, the specific people involved are not identified, and students only used the “Input” and “Output” aspects.

b. This FRAM from cycle 4, depicts the process of admitting a patient urgently. In addition to “Input” and “Output,” the students also used the aspects “Resource” and “Precondition”. They further indicated which steps are performed by which person using color coding.

c. The FRAM above from cycle 6 illustrates the discharge process of a patient going home. In this model, students did not use color coding but instead named the executor in each hexagon (highlighted in bold font). This approach allowed students to better visualize the involvement of different executors in a complex process.
